# Spatial selective auditory attention is preserved in older age but is degraded by peripheral hearing loss

**DOI:** 10.1038/s41598-024-77102-5

**Published:** 2024-10-31

**Authors:** Andrea Caso, Timothy D. Griffiths, Emma Holmes

**Affiliations:** 1 Department of Speech Hearing and Phonetic Sciences, Division of Psychology and Language Sciences, University College London, Chandler House, 2 Wakefield Street, London, WC1N 3PF UK; 2 Biosciences Institute, Newcastle University, Newcastle upon Tyne, UK; 3 Wellcome Centre for Human Neuroimaging, University College London, London, UK; 4 Human Brain Research Laboratory, University of Iowa, Iowa City, IA USA

**Keywords:** Attention, Ageing, Hearing, Speech perception, Human behaviour, Auditory system, Cognitive ageing

## Abstract

Interest in how ageing affects attention is long-standing, although interactions between sensory and attentional processing in older age are not fully understood. Here, we examined interactions between peripheral hearing and selective attention in a spatialised cocktail party listening paradigm, in which three talkers spoke different sentences simultaneously and participants were asked to report the sentence spoken by a talker at a particular location. By comparing a sample of older (*N* = 61; age = 55–80 years) and younger (*N* = 58; age = 18–35 years) adults, we show that, as a group, older adults benefit as much as younger adults from preparatory spatial attention. Although, for older adults, this benefit significantly reduces with greater age-related hearing loss. These results demonstrate that older adults with excellent hearing retain the ability to direct spatial selective attention, but this ability deteriorates, in a graded manner, with age-related hearing loss. Thus, reductions in spatial selective attention likely contribute to difficulties communicating in social settings for older adults with age-related hearing loss. Overall, these findings demonstrate a relationship between mild perceptual decline and attention in older age.

**Supplementary Information:**

The online version contains supplementary material available at 10.1038/s41598-024-77102-5.

## Introduction

How cognitive functions change with healthy ageing is a question of long-standing interest. Some cognitive abilities (e.g., working memory, visuo-spatial ability) appear to deteriorate with older age, whereas others (e.g., crystallised intelligence) improve^1^. Spatial attention is important for navigating everyday environments and social interactions, and previous studies have examined visual and auditory spatial attention in older listeners^1,2^. However, we do not fully understand how spatial attention is affected by sensory declines (e.g., in hearing and vision) that are prevalent in older age.

Some previous studies have attempted to isolate changes in attention from changes in performance related to the sensory processing of attended stimuli. For example, these studies have allowed older adults greater time to utilise an attentional cue or have equated baseline performance, and the results imply that age-related changes previously attributed to attention are not present after controlling for sensory processing^3–5^. However, these methods implicitly assume that changes in sensory processing are independent of attention—for example, by assuming that sensory decline alters the ‘perceptual level’ of processing, whereas changes in attention occur due to aspects of cognitive ageing that are unrelated to sensory decline^6,7^. Thus, we do not understand whether sensory processing affects the ability to deploy top-down attention^8^. Age-related declines in sensory processing may affect the allocation of attention—for example, by degrading the sensory representations that people utilise to orient attention or, conversely, through *increased* reliance on attentional processes to compensate for degraded sensory processing. Crucially, the consequences of sensory processing for the deployment of top-down attention could explain why previous studies on attentional changes in older age have found conflicting results^9^. In addition, sensory declines are extremely prevalent, so their effects on top-down attention are necessary for understanding cognitive processes in the vast majority of the ageing population, and could also provide insights into recently-established correlations between degraded peripheral function and risk of dementia^10,11^. Here, we assessed the relative contributions of age-related changes in attention that are dependent on sensory processing, and those that are independent of sensory processing, by explicitly modelling sensory effects on top-down attention.

We assessed sensory effects on attention in a cocktail-party listening paradigm, in which participants hear sentences spoken by multiple talkers and are visually cued to selectively attend to a sentence at a particular location. Crucially, hearing loss affects approximately 40% of people aged 55 and above^12^, and difficulties understanding speech in the presence of competing speech are not fully explained by measures of peripheral function, such as pure-tone audiometry^13,14^. Cocktail-party listening involves a variety of processes, including bottom-up sensory processing and top-down cognitive processes. How ageing affects speech intelligibility in cocktail-party listening has been well-studied, and some previous studies have attempted to separate the consequences of cognitive ageing on performance from the consequences of age-related hearing loss on performance^6,7,15–20^. Here, we were interested in specifically studying attention during cocktail-party listening to examine possible effects of sensory processing on top-down attention: We included an instructive visual cue that participants could use to orient attention to an upcoming talker, which allowed us to measure endogenous attentional orienting (a type of ‘covert’ attention^21^, which has also been used to describe situations in which a participant changes the focus of visual attention without moving their eyes) during this task. By comparing conditions in which the acoustic and other cognitive demands of the task are equal, but manipulating the duration of time that participants have to orient attention, we could examine how attentional orienting is affected by ageing and by hearing loss, separately from the consequences of hearing loss for the perceptual processing of auditory stimuli.

For young adults with normal hearing, orienting spatial attention is known to improve speech intelligibility in cocktail-party paradigms^22,23^, which appears to be underpinned by preparatory activity in fronto-parietal regions of cortex during the cue-target interval^24,25^ that may facilitate performance^26^. However, people with sensorineural hearing loss may not utilise spatial attention to the same extent. For example, Best et al.^27^ found that young adults (19–42 years) with hearing loss gained a significantly smaller improvement in intelligibility from a visual cue that informed them about the location of an upcoming target talker, compared to young adults with normal hearing. In addition, Holmes et al.^28^ found that EEG responses elicited by a visual cue for attention were weaker in children (aged 7–16 years) with than those without early-onset sensorineural hearing loss. Both studies recruited participants who did not have visual comorbidities, so degraded visual processing of the cue is an unlikely explanation for differences between groups^29^. Crucially, these studies of *preparatory* spatial attention allow auditory attention to be measured without confounding differences in peripheral auditory processing between people with and without hearing loss.

Yet, given the aforementioned studies^27,28^ tested young participants, we do not understand how *age-related* hearing loss affects preparatory spatial attention. On one hand, poorer spatial acuity may reduce the ability to orient spatial attention: Hearing loss degrades spectrotemporal resolution^30–33^, which can affect binaural and monaural spatial cues^34^. Reduced spatial acuity could cause difficulty orienting selective attention to filter out distracting sounds, effectively broadening the focus of spatial attention and impairing sound segregation^35,36^. Given that age-related hearing loss increases the minimal audible angle for discriminating spatial locations (to about 4 degrees, compared to approximately 1 degree in people without hearing loss^37^), then this explanation predicts that hearing loss at any age should degrade spatial attention, so older adults with age-related hearing loss should have weaker spatial attention than (older and younger) adults with normal hearing. Broadly speaking, these changes could be considered as a “central effect of peripheral pathology”, along the lines suggested by Willott (1996)^38^ and reflected in a variety of neuroimaging studies^39–41^; although, Willott (1996) described far-reaching changes to processing in the central auditory system, whereas the current explanation proposes specific differences related to auditory spatial processing. Crucially, this explanation differs from theories that predict differences in attention due to cognitive ageing (e.g., previously termed “central effects of biological ageing” by Willott^38^) because, under the current explanation, a hearing loss (rather than ageing) is the cause of differences in spatial attention. This explanation also differs from previous descriptions of “central presbycusis”^42^, which assumes differences in central auditory processing that are separate from those related to peripheral hearing loss. It also differs from accounts based on changes to the ‘perceptual level’ of processing following hearing loss^43,44^, as these accounts tend to assume that differences can be explained by changes to the bottom-up processing of auditory stimuli (e.g., reduced audibility or changes in suprathreshold processing, such as frequency or temporal processing) rather than (associated) changes to top-down processing.

On the other hand, spatial attention may not scale with spatial acuity. In the aforementioned studies of auditory attention, stimuli were separated by much greater angles (i.e., ≥ 15 degrees) than the minimum audible angle. Thus, reduced spatial acuity may not affect spatial attention at these angles of spatial separation, so spatial attention may be preserved in older adults with age-related hearing loss. Another possible explanation for why spatial attention could scale with spatial acuity is through an indirect route: Possibly, people with hearing loss learn that directing spatial attention has limited utility if spatial cues are degraded by hearing loss—yet, it is also possible that this explanation could apply only to *early-onset* hearing loss and not to older adults with age-related hearing loss, who have considerable experience directing attention to sounds before they acquired hearing loss. Therefore, based on either of these arguments, older adults might deploy spatial attention to a similar extent as adults with normal hearing. Under the latter explanation, older adults with age-related hearing loss may even deploy spatial attention to a *greater* extent to help compensate for sensory decline. While compensatory accounts have been proposed as an explanation for why semantic context may be used to assist speech perception to a greater extent in older adults^41,45,46^, it is unclear whether compensation would apply to endogenous attention in adults with age-related hearing loss.

Here, we compared the extent to which older and younger adults use centrally-presented visual spatial cues (presented on a computer screen) to improve speech intelligibility in a cocktail-party listening task. We recruited participants with mixed degrees of hearing loss and measured each participant’s audiometric thresholds and spatial discrimination thresholds to examine whether preparatory spatial attention depends, in a graded manner, on the degree of hearing loss and spatial resolution—to separate changes to attention that co-occur with changes in sensory processing from changes to attention that change with older age independently of peripheral function. Given that speech intelligibility in noisy environments has been hypothesised to relate to overall cognitive ability^47,48^, we also included a visual matrix reasoning test (often used to assess fluid intelligence^49^), to examine whether performance on this visual cognitive test relates to speech intelligibility overall, or to the benefit that each participant gains from orienting endogenous spatial attention.

## Results

### Older and younger adults gain a similar benefit to the accuracy of speech intelligibility from advance spatial cues

**Fig. 1:**
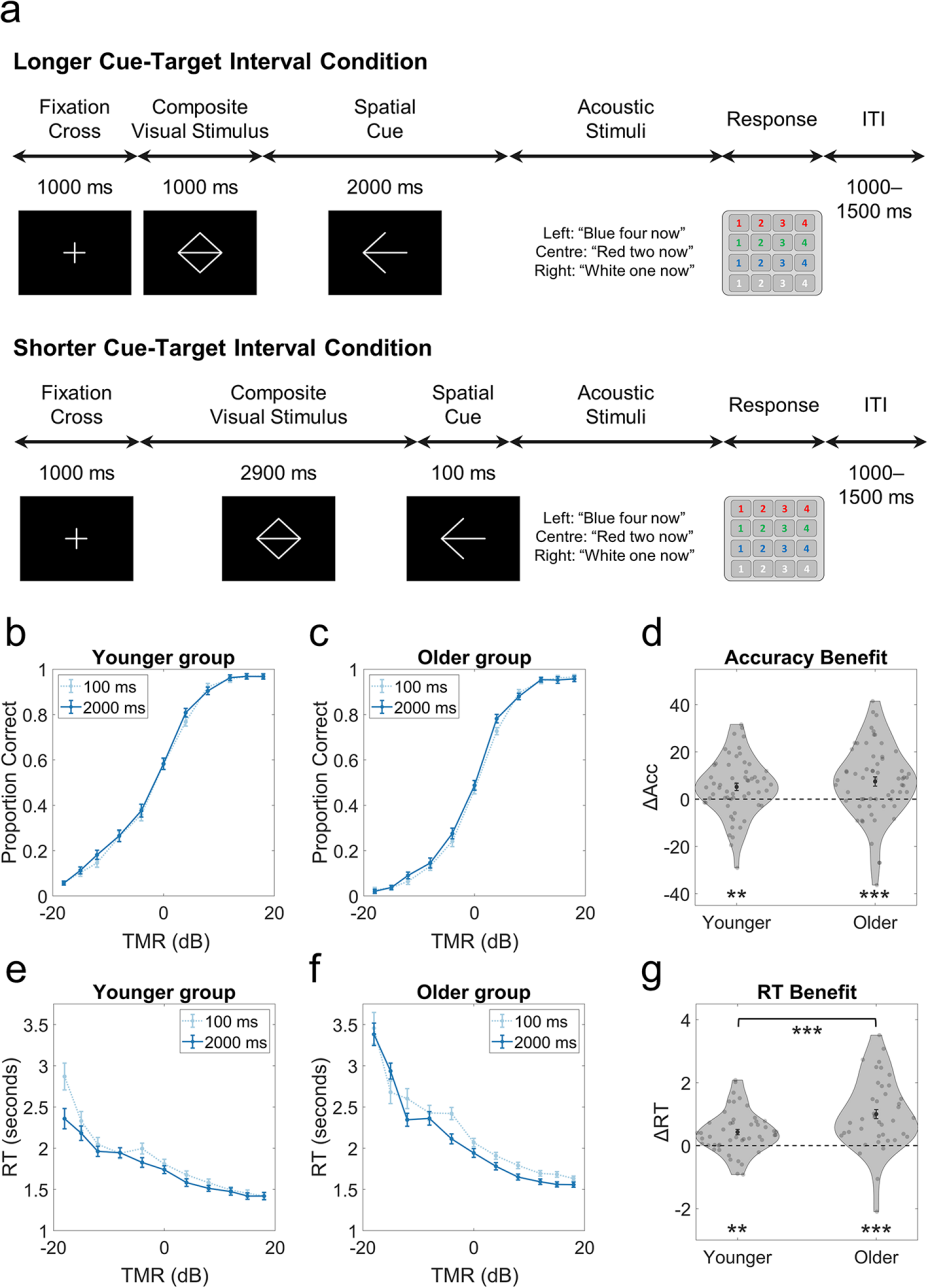
Experimental design and performance across groups. (**a**) Schematic of the trial structure for the longer and shorter cue-target interval conditions. The visual cue was an arrow that either pointed to the left (as depicted) or to the right. The three acoustic phrases were spoken by different talkers and simulated to originate from left, central, and right locations using interaural time differences (ITDs). ITI: Inter-trial interval. (**b**) Proportion of correct responses in the spatial attention task for the younger group, on average, at each cue-target interval condition (100, 2000 ms) and each target-to-masker ratio (TMR) condition. (**d**) Proportion of correct responses in the spatial attention task for the older group. (**d**) The benefit to accuracy from preparatory spatial attention, calculated as the difference in accuracy between the longer and shorter cue-target interval conditions. Black dots and black bars indicate the means and standard errors in each group, grey dots indicate results from individual participants, and the width of the violin plots indicates the distribution of results across participants. (**e**) Reaction times (RTs) measured from the onset of acoustic stimuli for the younger group, on average, across conditions. Error bars show ± 1 standard error of the mean. (**f**) RTs for the older group. (**g**) The benefit to RTs from preparatory spatial attention.

We measured accuracy and reaction times (RTs) in a spatialised cocktail party listening paradigm (Fig. 1a), in which three talkers spoke different sentences simultaneously from different locations. Participants were asked to report the sentence spoken by a talker at a particular location, which was indicated by a visual spatial cue that was presented either 2000 or 100 ms before the acoustic stimuli began. We varied the target-to-masker ratio (TMR)—in other words, the difference in level between the target and competing sentences—between − 18 and + 18 decibels, so that we could characterise the psychometric functions. This allowed us to measure differences between cue-target interval conditions and groups across a range of performance levels, given that the degree of benefit may depend on the specific TMR and the level of performance^50,51^. Responses were recorded as correct if both the colour and number words were correctly identified from the target sentence.

First, we calculated the proportion of correct responses at each TMR, separately for the longer and shorter cue-target interval conditions, and separately for each group. Figure 1b–c illustrates the proportion correct across groups, TMRs, and cue-target intervals. As expected, accuracy improved with increasing TMR, *F*(10, 1170) = 2038.3, *p* < .001, *ω*_*p*_^2^ = 0.89, 95% CI = [0.88, 0.90]. Accuracy was greater for the younger group (mean = 0.557, standard deviation = 0.104) than for the older group (mean = 0.505, standard deviation = 0.077), *F*(1, 117) = 9.60, *p* < .001, *ω*_*p*_^2^ = 0.07, 95% CI = [0.01, 0.18]. There was also greater accuracy in the 2000-ms cue-target interval condition (mean = 0.535, standard deviation = 0.098) than the 100-ms cue-target interval condition (mean = 0.525, standard deviation = 0.093), *F*(1, 1170) = 23.2, *p* < .001, *ω*_*p*_^2^ < 0.01, 95% CI = [0.00, 0.05].

The interaction between Group and TMR was significant, *F*(10, 1170) = 5.67, *p* < .001, *ω*_*p*_^2^ = 0.02, 95% CI = [0.00, 0.03], indicating that the difference in accuracy between the two age groups differed across TMRs. Follow-up independent-samples *t*-tests (with Bonferroni correction for multiple comparisons, ‘*p*_*bonf*_*’*) showed that the younger group had greater accuracy than the older group at -8 dB TMR [*t*(117) = 5.00, *p*_*bonf*_ < 0.001, *d*_*s*_ = 0.92, 95% CI = 0.54, 1.29], -4 dB TMR [*t*(117) = 4.23, *p*_*bonf*_ = 0.007, *d*_*s*_ =. 78, 95% CI = 0.40, 1.15], and 0 dB TMR [*t*(117) = 3.93, *p*_*bonf*_ = 0.023, *d*_*s*_ = 0.73, 95% CI = 0.35, 1.09], but there were no significant differences at the other TMRs, *t*(117) ≤ 3.51, *p*_*bonf*_ ≥ 0.113, *d*_*s*_ ≤ 0.65.

The interaction between Cue-Target Interval and TMR was significant, *F*(10, 1170) = 7.91, *p* < .001, *ω*_*p*_^2^ = 0.01, 95% CI = [0.00, 0.01], indicating that the difference in accuracy between the two cue-target interval conditions differed across TMRs. Follow-up paired-samples *t*-tests (with Bonferroni correction for multiple comparisons) showed that accuracy was greater in the longer cue-target interval condition than the shorter cue-target interval condition at -12 dB TMR [*t*(118) = 4.67, *p*_*bonf*_ < 0.001, *d*_*z*_ = 0.43, 95% CI = 0.24, 0.61], -4 dB TMR [*t*(118) = 3.92, *p*_*bonf*_ = 0.021, *d*_*z*_ = 0.36, 95% CI = 0.17, 0.54], and 4 dB TMR [*t*(118) = 7.20, *p*_*bonf*_ < 0.001, *d*_*z*_ = 0.66, 95% CI = 0.46, 0.86], but there were no significant differences at the other TMRs, *t*(118) ≤ 2.58, *p*_*bonf*_ ~ 1.00, *d*_*z*_ ≤ 0.26.

We found no significant interaction between Cue-Target Interval and Group, *F*(1, 117) = 0.51, *p* = .477, *ω*_*p*_^*2*^ < 0.01, 95% CI = [0.00, 1.00], and no significant three-way interaction between Group, Cue-Target Interval, and TMR, *F*(10, 1170) = 0.59, *p* = .824, *ω*_*p*_^2^ < 0.01, 95% CI = [0.00, 1.00].

To compare the benefit from preparatory spatial attention between groups, we first calculated a metric to assess the benefit in each participant. For each participant in each cue-target interval condition, we fitted a sigmoid curve to proportion-correct scores across TMRs using a generalised linear model with a logistic link function (MATLAB function *glmfit* with argument ‘*logit’*). Next, we calculated the area under the curve for each cue-target interval condition. We took the difference in the area under the curve (ΔAcc) between the longer and shorter cue-target interval conditions as the benefit to speech intelligibility for each participant (with negative values indicating a decrement to speech intelligibility from the longer spatial cue). The advantage of using this metric (rather than comparing performance between cue-target interval conditions at individual TMRs) is that, by considering responses across a wide range of TMRs, we avoid ceiling and floor effects, and account for differences in overall speech intelligibility among individuals (and, therefore, between groups).

Figure 1d illustrates the accuracy benefit from preparatory spatial attention (ΔAcc) for the two groups. One-sample *t*-tests showed a significant benefit (i.e., greater than zero) in both younger adults, *t*(57) = 3.23, *p* = .002, *d*_*z*_ = 0.42, 95% CI = [0.15, 0.69], and older adults, *t*(60) = 3.82, *p* < .001, *d*_*z*_ = 0.49, 95% CI = [0.22, 0.75]. An independent samples *t*-test showed that the magnitude of the benefit did not differ between groups, *t*(117) = 0.91, *p* = .367, *d*_*z*_ = 0.17, 95% CI = [-0.19, 0.53]. Given one of the main goals of this study was to compare the magnitude of the benefit from preparatory spatial attention between age groups, we followed up this non-significant difference by conducting a Bayesian equivalence test (using a Cauchy distribution centred around zero as a prior, which is the default prior in JASP). This allowed us to assess the evidence that the difference between groups is either identical or else has a small effect size (between − 0.10 and 0.10). The results showed a Bayes Factor of 3.50, which provides some evidence to support a similar-magnitude benefit to speech intelligibility from preparatory spatial attention in younger and older adults.

### Older adults gain a greater benefit to reaction times from advance spatial cues than do younger adults

Figure 1e–f illustrates RTs in the spatial attention task across Groups, TMRs, and Cue-Target Intervals. As expected, RTs were faster with increasing TMR, *F*(7, 609) = 180.7, *p* < .001, *ω*_*p*_^2^ = 0.31, 95% CI = 0.24, 0.36. Overall, the younger group had faster responses (mean = 1.68 s, standard deviation = 0.70) than the older group (mean = 2.06 s, standard deviation = 0.34), *F*(1, 87) = 9.36, *p* = .003, *ω*_*p*_^2^ = 0.07, 95% CI = 0.00, 0.19. RTs were faster in the 2000-ms cue-target interval condition (mean = 1.81 s, standard deviation = 0.34) than the 100-ms cue-target interval condition (mean = 1.91 s, standard deviation = 0.39), *F*(1, 87) = 40.6, *p <* .001, *ω*_*p*_^2^ = 0.02, 95% CI = 0.00, 0.11]. Although, we also found significant two-way interactions, which should be considered when interpreting the main effects.

The interaction between Group and TMR was significant, *F*(7, 609) = 4.53, *p <* .001, *ω*_*p*_^2^ = 0.01, 95% CI = [0.00, 0.02], showing that age group had different effects on RTs depending on the TMR. Follow-up independent samples *t*-tests (with Bonferroni correction for multiple comparisons) indicated that this interaction was driven by slower RTs in the older group compared to the younger group at -8 dB TMR, *t*(94) = 4.63, *p*_*bonf*_ < 0.001, *d*_*s*_ = 0.96, 95% CI = [0.52, 1.37], and at -4 dB TMR, *t*(105) = 4.18, *p*_*bonf*_ = 0.006, *d*_*s*_ = 0.82, 95% CI = [0.41, 1.20]; whereas, there were no significant differences in RTs between groups at the other TMRs, *t*(≥ 85) ≤ 2.41, *p*_*bonf*_ ~ 1.00, *d*_*s*_ ≤ 0.47.

A significant interaction between Cue-Target Interval and TMR, *F*(7, 609) = 5.03, *p* < .001, *ω*_*p*_^2^ = 0.01, 95% CI = [0.00, 0.01] indicated that the difference in RTs between the two cue-target interval conditions differed across TMRs. Paired-sample t-tests (with Bonferroni correction for multiple comparisons) showed faster RTs in the longer cue-target interval condition than the shorter cue-target interval condition at -4 dB TMR [*t*(106) = 7.35, *p*_*bonf*_ < 0.001, *d*_*z*_ = 0.71, 95% CI = 0.50, 0.92] and 4 dB TMR [*t*(108) = 3.84, *p*_*bonf*_ = 0.016, *d*_*z*_ = 0.37, 95% CI = 0.17, 0.56], but no significant difference at the other TMRs, *t*(88) ≤ 3.39, *p*_*bonf*_ ≥ 0.090, *d*_*z*_ ≤ 0.27.

We also found a significant interaction between Group and Cue-Target Interval, *F*(1, 87) = 6.91, *p* = .010, *ω*_*p*_^2^ < 0.01, 95% CI = [0.00, 0.07]. Follow-up independent samples *t*-tests (with Bonferroni correction for multiple comparisons) showed a significant difference in RTs between groups at the shorter cue-target interval, *t*(87) = 3.55, *p*_*bonf*_ = 0.004, *d*_*z*_ = 0.76, 95% CI [0.32, 1.18], but no significant difference at the longer cue-target interval, *t*(87) = 2.43, *p*_*bonf*_ = 0.103, *d*_*z*_ = 0.52, 95% CI = [0.09, 0.94].

The three-way Group x Cue-Target Interval x TMR interaction was not significant, *F*(7, 609) = 0.412, *p* = .895, *ω*_*p*_^2^ < 0.01, 95% CI [0.00, 1.00].

Similar to accuracy, we calculated a metric to assess the benefit to RTs from preparatory spatial attention across TMRs, so we could compare the benefit to RTs between groups. For each participant in each cue-target interval condition, we fitted a polygonal line to the RT data across TMRs from − 8 dB to + 18 dB TMR. We then calculated the difference (ΔRT) between the fitted lines for the longer and shorter cue-target interval conditions for each participant, which we took as the RT benefit (with negative values indicating a decrement to—i.e., an increase in—RTs from the longer spatial cue).

Figure 1g illustrates the RT benefit from preparatory spatial attention (ΔRT) for the two groups. One-sample *t*-tests showed a significant benefit in both younger adults, *t*(57) = 4.74, *p* = .002, *d*_*z*_ = 0.62,, 95% CI = [0.34, 0.90] and older adults *t*(60) = 7.49, *p* < .001, *d*_*z*_ = 0.96, 95% CI = [0.65, 1.26]. An independent-samples *t*-test revealed that the RT benefit was significantly larger in the older group than in the younger group, t(117) = 3.62, *p* < .001, *d*_*z*_ = 0.67, 95% CI = [0.29, 1.03].

### Individual differences in task performance relate to spatial acuity, matrix reasoning scores, and hearing sensitivity

Given we were interested in individual differences among participants, we conducted several analyses to identify relationships among tasks. To examine how performance relates to hearing loss, we included pure-tone audiometric thresholds at 4–8 kHz, averaged across the two ears (hereafter, ‘Audiogram’), This measure of high-frequency hearing captures the typical changes in older adults (for more detail, see Methods). We also included ITD thresholds (i.e., spatial discrimination thresholds corresponding to the minimal audible angle), and age-scaled matrix reasoning scores^52^ (hereafter, ‘WAIS-MR’). For these analyses, we examined relationships among variables in the younger and older groups separately.

First, given that most previous studies examining relationships among variables have used raw speech intelligibility scores^35,47^ (rather than the benefit from a longer cue-target interval), we investigated how individual differences in overall accuracy and RTs (collapsed across TMRs) on the spatial attention task relates to the other variables. For each group, we examined correlations between 6 pairs of variables (Audiogram, ITD Threshold, and WAIS-MR score with overall Accuracy and with overall RTs in the spatial attention task). The significance threshold was adjusted for multiple comparisons using the Bonferroni correction (*p* < .05 / 6 = 0.0083).

**Fig. 2:**
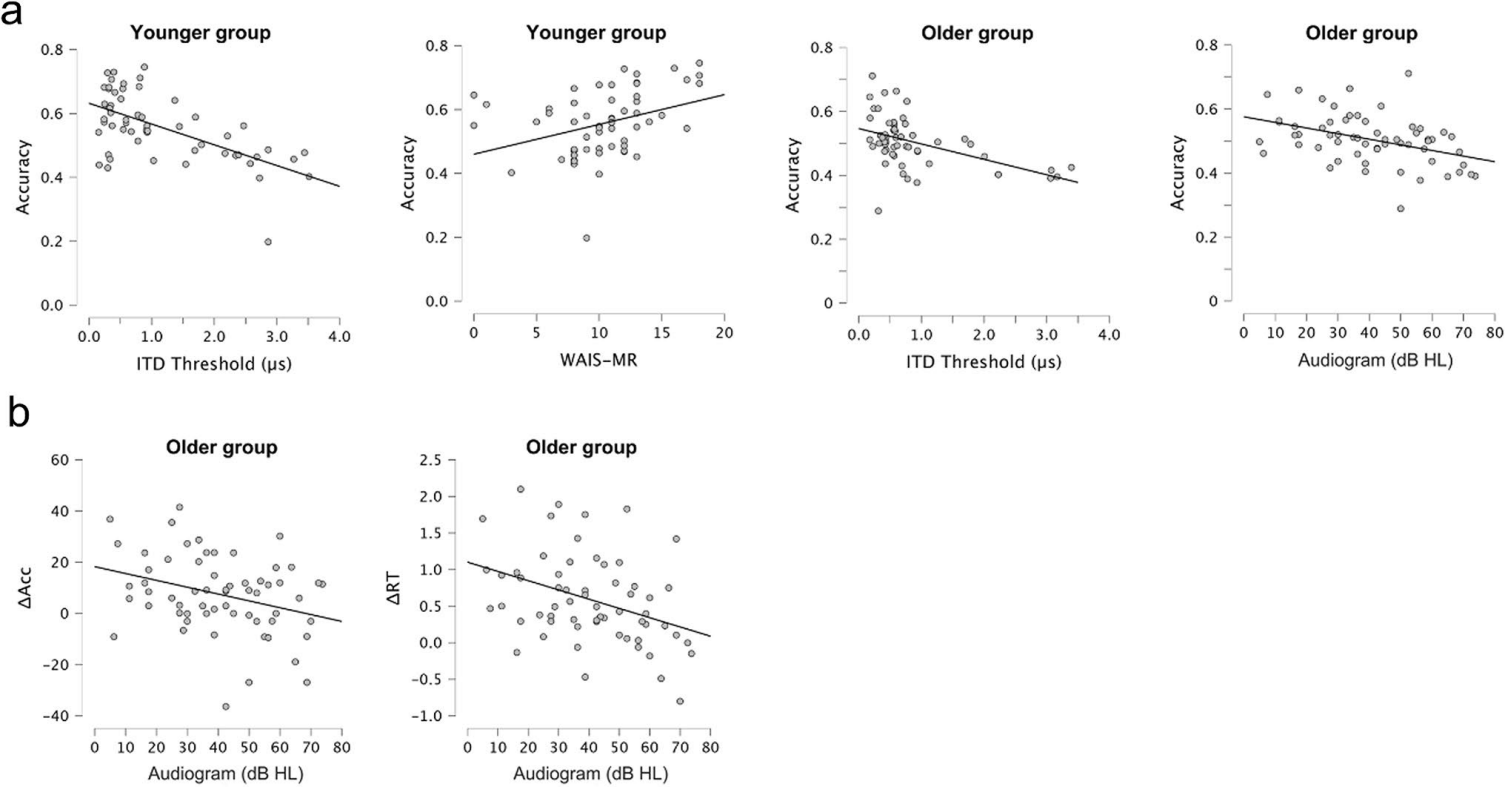
Scatter plots showing significant relationships between variables. (**a**) Significant relationships with overall accuracy in the spatial attention task: ITD Thresholds and WAIS-MR age-scaled scores for the younger group; ITD Thresholds and the Audiogram for the younger group. Individual points each represent one participant, and the solid lines display the lines of best fit. (**b**) Significant relationships between the Audiogram and the benefit from the longer cue-target interval in the older group, both for the benefit to accuracy (ΔAcc) and the benefit to reaction times (ΔRT). Positive values indicate better performance (i.e., greater accuracy or faster RTs in the longer cue-target interval condition).

For the younger group, better (i.e., smaller) ITD Thresholds were associated with better Accuracy in the spatial attention task (*ρ* = − 0.49, *p* < .001; Fig. 2a). In addition, better WAIS-MR scores were associated with better Accuracy (*ρ* = 0.43, *p* < .001; Fig. 2a). None of the other correlations with Accuracy or RTs were significant (see Table 1). To test whether WAIS-MR and ITD Threshold explained separate variance in Accuracy in the younger group, we entered these variables into a stepwise linear regression. The results showed that WAIS-MR and ITD Threshold made significant independent contributions to predicting Accuracy in the spatial attention task (model containing ITD Threshold only: *R*^*2*^ = 0.37, *p* < .001; model containing ITD Threshold and WAIS-MR: *R*^*2*^ = 0.46, *R*^2^ change = 0.09, *p* change = 0.005).

**Table 1:** Spearman’s rho (*ρ*) and associated *p*-values for relationships with overall accuracy and reaction times (RTs) in the spatial attention task.

Variable 1	Variable 2	Younger group		Older group	
		ρ	*p*	ρ	*p*
Audiogram	Accuracy	− 0.14	0.283	**− 0.43**	**< 0.001**
ITD threshold	Accuracy	**− 0.49**	**< 0.001**	**− 0.49**	**< 0.001**
WAIS-MR	Accuracy	**0.43**	**< 0.001**	0.30	0.020
Audiogram	RTs	0.13	0.319	0.26	0.044
ITD threshold	RTs	0.28	0.033	0.06	0.683
WAIS-MR	RTs	0.02	0.870	0.32	0.013

Relationships that surpass the Bonferroni-corrected significance threshold (*p* < .0083) are indicated with bold font.

For the older group, we found a significant correlation between ITD Thresholds and Accuracy, *ρ* = − 0.49, *p* < .001 (Fig. 2a), like for the younger group. We also found a significant correlation between the Audiogram and Accuracy, *ρ* = − 0.43, *p* < .001 (Fig. 2a). None of the other correlations were significant (see Table 1). Including both ITD Threshold and the Audiogram in a stepwise linear regression showed that these two variables made significant independent contributions to predicting Accuracy in the older group (model containing ITD Threshold only: *R*^*2*^ = 0.23, *p* < .001; model containing ITD Threshold and Audiogram: *R*^*2*^ = 0.29, *R*^2^ change = 0.06, *p* change = 0.037).

### Individual differences in orienting auditory spatial attention among older adults are predicted by hearing sensitivity

While the previous analyses examined individual differences in overall performance, we were primarily interested in how the *benefit* from preparatory spatial attention varied across participants. We, therefore, examined relationships between the benefit to accuracy (ΔAcc) and benefit to RTs (ΔRT) from the longer cue-target interval with the Audiogram, ITD Threshold, and WAIS-MR score.

In the younger group, none of the correlations were significant, for either the benefit to accuracy or to RTs (Table 2).

**Table 2:** Spearman’s rho (*ρ*) and associated *p*-values for relationships with the benefit to accuracy (ΔAcc) and to reaction times (ΔRT) from preparatory spatial attention.

Variable 1	Variable 2	Younger group		Older group	
		ρ	*p*	ρ	*p*
Audiogram	ΔAcc	− 0.11	0.419	**− 0.27**	**0.037**
ITD threshold	ΔAcc	− 0.25	0.067	− 0.24	0.065
WAIS-MR	ΔAcc	− 0.18	0.184	0.04	0.777
Audiogram	ΔRT	− 0.03	0.849	**− 0.39**	**0.002**
ITD threshold	ΔRT	− 0.10	0.462	0.12	0.357
WAIS-MR	ΔRT	− 0.20	0.136	− 0.14	0.294

Relationships that surpass the significance threshold (*p* < .05) are indicated with bold font

In the older group, there were no significant correlations with ITD Threshold or WAIS-MR scores (Table 2). However, the Audiogram correlated significantly with both the benefit to accuracy (*ρ* = − 0.27, *p* = .037; Fig. 2b) and the benefit to RTs (*ρ* = 0.39, *p* = .002; Fig. 2b). The direction of these correlations indicates that greater hearing loss reduces the benefit from preparatory spatial attention, both when measured in terms of the improvement in accuracy and the improvement in RTs. We did not correct for multiple comparisons in this analysis, because these were planned comparisons related to the main hypothesis. Although, it is interesting to note that the correlation with ΔRT would remain significant after Bonferroni correction. To assess whether this relationship was a by-product of older age, we ran a follow-up partial Spearman’s correlation controlling for age in years. The correlation between the Audiogram and ΔRT remained significant after controlling for age (*ρ* = 0.44, *p* = .001).

Given that the younger and older groups showed different relationships between the Audiogram and the benefit from preparatory spatial attention, we conducted follow-up (two-tailed) bootstrapping analyses to compare the Spearman’s correlation coefficients between the two groups. The correlation between the Audiogram and ΔRT was significantly greater in older than younger adults [observed Δ*ρ* = 0.40, *p* = .002; null distribution mean Δ*ρ* < 0.01, 95% CI = − 0.27, 0.26]. Although, there was no significant difference in correlation coefficients between the two groups for the relationship between the Audiogram and ΔAcc [observed Δ*ρ* = 0.19, *p* = .083; null distribution mean Δ*ρ* < 0.001, 95% CI = − 0.25, 0.26].

## Discussion

We sought to disentangle how orienting spatial attention relates to (cognitive) ageing, in general, and to sensory declines that occur with older age. We found that the benefit to speech perception from preparatory spatial attention is at least as large in older as in younger adults, as a group (Fig. 1d). Thus, consistent with previous work on visual endogenous attention^3–5^, people aged 55 and above generally retain the capacity to orient auditory spatial attention. Nevertheless, our results demonstrate effects of sensory decline on attentional orienting, which have not been explicitly modelled in previous studies, that are crucial for understanding attention in older age. We found that sensory processing affected the magnitude of the benefit from preparatory spatial attention: Greater age-related hearing loss progressively reduced the benefit (Fig. 2b). Thus, while spatial selective auditory attention is preserved in older age, it is degraded by age-related hearing loss. Given that more than 40% of adults aged 55 and above experience clinically significant declines in hearing sensitivity^12^, our findings highlight the importance of considering peripheral sensory processing as a crucial predictor of attentional orienting in older age.

Our results extend previous work showing impaired preparatory spatial attention in children with early-onset hearing loss^28^, by demonstrating that this also applies to hearing loss occurring much later in the lifespan. In addition, our results indicate that the ability to orient spatial attention is not all-or-none but rather varies in magnitude across individuals depending on their degree of hearing loss. In addition to the difficulties understanding speech posed by reduced audibility, declines in spatial attention may make it more difficult for individuals to perceptually segregate talkers in situations with multiple competing talkers (which is supported by different patterns of errors between older and younger adults: see Supplemental Fig. 1). Our finding is particularly noteworthy because most of our sample have audiometric thresholds that would not lead to a clinical diagnosis of hearing loss. Thus, even small variations in hearing sensitivity impact endogenous spatial attention, which could help to explain why many middle-aged adults report difficulties understanding speech in noisy environments, despite not meeting the criteria for a hearing-loss diagnosis^53,54^.

In general, our findings oppose a mechanism by which orienting spatial attention compensates for deteriorations in sensory processing (i.e., audiometric thresholds), because under this explanation we would expect a *greater* benefit from preparatory spatial attention in people who had more age-related hearing loss, whereas our results showed the opposite pattern (i.e., a smaller benefit from preparatory spatial attention in people who had more age-related hearing loss; Fig. 2b). Instead, our results are consistent with a more nuanced explanation that may involve multiple mechanisms.

The first mechanism relates to the enhancement of the RT benefit from preparatory spatial attention in older age (Fig. 1g), which we found was unrelated to spatial acuity (Table 2) and could instead compensate for other processes that change with age—such as processing speed^55–57^ and/or response speed^58,59^ (for a review, see Ebaid & Crewther^60^). This compensatory explanation is consistent with our finding that RTs were significantly slower in older than younger adults in the shorter cue-target interval condition but did not differ significantly between groups in the longer cue-target interval condition. Alternatively, we might have only found a greater benefit to RTs because RTs in the shorter cue-target interval condition were slower in older adults and, therefore, their RTs had greater room to improve. Under the latter argument, no compensatory mechanism would be required to explain our results.

A second mechanism is needed to account for the finding that declines in sensory processing *reduce* the benefit from preparatory spatial attention. This pattern of results cannot be explained by the stimuli simply being inaudible for some participants, because our measure of preparatory spatial attention was calculated as a difference in performance between two cue-target interval conditions that contained identical acoustic stimuli; also, the difference was measured across a range of TMRs that spanned the full range of performance between floor and ceiling. Interestingly, although overall speech intelligibility in our spatial attention task depended on a listener’s spatial acuity (consistent with Dai et al.^35^), we found no evidence that the magnitude of the benefit from preparatory spatial attention was related to spatial acuity. This implies that the mechanism by which age-related hearing loss affects preparatory spatial attention is unlikely to be due to reduced spatial acuity and, therefore, the relationship between the benefit from preparatory spatial attention and audiometric thresholds may be due to other effects of hearing loss. Based on previous literature^61,62^, an alternative possibility was that the magnitude of the benefit could be related to more generic cognitive changes associated with hearing loss that could be assessed by performance in the visual WAIS matrix reasoning task. However, we found no evidence for a relationship between the benefit from preparatory spatial attention and WAIS matrix reasoning scores—compatible with the results of Gatehouse and Akeroyd^63^, which showed no relationship between the benefit from attentional cues in a cocktail-party listening paradigm and performance on the Test of Everyday Attention (which included both visual and auditory tests). Thus, a new mechanism may be required to explain the relationship we found between preparatory spatial attention and audiometric thresholds.

The specific mechanism that explains the relationship between preparatory spatial attention and audiometric thresholds is unclear, but there are several possibilities. Previous work has characterised the knock-on consequences of peripheral hearing loss for the central auditory pathway, including brainstem and cortex^38,64–66^; possibly, differences in attentional orienting could reflect structural and functional^39,40^ changes to the central auditory pathway. These types of changes might degrade the ability to deploy top-down attention, either directly by affecting the neural substrates of preparatory spatial attention (possibility, similar to the types of links proposed to occur between hearing loss and working memory^67^) or indirectly due to limited cognitive resources—for example, due to greater cognitive demand^68^, load^69^, or effort^70^ that arises as a result of hearing loss (which is compatible with resource-allocation frameworks^41^). Given that preparatory spatial attention would be considered a form of “controlled processing” that requires effort under “automatic/controlled processing theory”^71,72^, greater effort due to (the consequences of) hearing loss could mean that insufficient resources are available to deploy preparatory spatial attention. Although, a different way of thinking about functional changes is that they can be adaptive, allowing participants to use different neural processes to achieve the same overall goal—which, for the purposes of the current task, could be maintaining the best possible speech understanding in everyday environments. For example, some studies suggest that reduced audibility encourages greater reliance on semantic context^46,73^—so, potentially, functional pathways could reorganise in a way that increases reliance on semantic context during cocktail-party listening but, as a side-effect, decreases reliance on other processes that can assist cocktail-party listening, such as spatial orienting. This type of functional reorganisation could be thought of—in psychological terms—as “reallocating cognitive resources”^44^ or a change in listening “strategy”^74^ away from relying on spatial orienting towards using other types of information (e.g., semantic context).

The idea that sensory processing affects attentional orienting is broadly consistent with hypotheses that age-related changes in sensory processing affect other aspects of auditory cognition, including working memory^75^ and processing speed^76^. Although, the neural underpinnings might differ when considering different aspects of auditory cognition. An interesting direction for future work would be to compare how age-related changes in sensory processing (separate from cognitive ageing) affect the neural processes involved in various aspects of auditory cognition.

To calculate the benefit to speech intelligibility from preparatory spatial attention (Figs. 1d and g and 2b and b), we essentially summed the benefit across TMRs. Although, as we might expect, the difference between cue-target intervals was greatest in the mid-to-low range of the TMRs we tested (Fig. 1b, c,e, f). By design, we aimed to capture floor and ceiling performance at the lowest and highest TMRs we tested, so that we could measure the full psychometric function for both groups. Yet, the difference in performance between the longer and shorter cue-target interval conditions was greatest at TMRs between − 12 and + 4 dB TMR. Therefore, performance at these TMRs is primarily responsible for the benefits we observed. We do not consider these particular TMRs to be special in any way and expect the effects were greatest at these TMRs because they were towards the middle of the psychometric function in the current experimental context. Crucially, the advantage of calculating the benefit across the psychometric function is that our results are not specific to performance at any one TMR, or to any particular level of performance.

The magnitude of the difference in RTs (between the shorter and longer cue-target interval conditions in the younger group at 0 dB TMR)—of approximately 70 ms—is smaller than in Holmes et al.^22^, in which the equivalent difference in RTs was approximately 200 ms. A possible reason for this difference is that the current experiment simulated spatial locations using ITDs, whereas Holmes et al.^22^ presented sounds in the free field (i.e., using loudspeakers). Deng et al.^77^ compared auditory spatial attention using different spatialisation simulations and found that, on average, accuracy was worse when spatial location was simulated using ITDs than when it was simulated using head-related transfer functions, which provide richer spatial content; also, they found that neural signatures of auditory attention were weaker when only ITDs were used. Therefore, our results may underestimate the extent to which younger and older participants benefit from preparatory spatial attention.

Most previous studies that have attempted to separate the consequences of cognitive ageing from age-related hearing loss have measured performance during cocktail party listening, rather than examining the benefit of instructive visual cues^6,7,15–18^. Given that many different processes contribute to performance during cocktail party listening, including audibility and suprathreshold auditory perceptual abilities (e.g., binaural processing, spectrotemporal processing), individual differences in these processes could contribute to differences previously attributed to age or hearing loss. Consistent with previous research, we found that overall speech intelligibility (on average, across all of the cue-target interval and TMR conditions we tested) was worse for older listeners who had poorer hearing sensitivity^78^ or poorer spatial acuity^35^ (Fig. 2a). The relationship with spatial acuity persisted in both older and younger adults, implying that even small differences in spatial acuity among young adults without hearing loss can help to predict their ability to understand speech in spatialised settings. Also, WAIS Matrix Reasoning scores covaried with overall performance (Fig. 2a; Supplemental Fig. 2). We assume this relationship is likely related to fluid intelligence, rather than to the spatial component of the matrix reasoning task, given that a previous study found a relationship between matrix reasoning and speech intelligibility in a non-spatialised sentence-in-noise task^79^.

In conclusion, while endogenous auditory spatial orienting is generally preserved in older age, it is degraded by age-related hearing loss—demonstrating that even small variations in hearing sensitivity with age impact endogenous attention. Interestingly, we found that the degradation of auditory spatial attention is not explained by poorer spatial acuity associated with hearing loss. Given that sensory decline is extremely prevalent among older adults, the impact of sensory changes on top-down attention is more likely to be the norm than the exception, and is important for predicting real-world perception among older populations.

## Methods

### Participants

We recruited 61 younger adults aged 18–35 years and 64 older adults aged 55–81 years. Of these, we excluded six participants (three from each group): one younger participant had recent ear surgery, two (1 older, 1 younger) withdrew part-way through the study, two (1 older, 1 younger) did not follow the task instructions (because, after the experiment, they told us that they ignored the attentional cues during the spatial attention task), and one older participant had below-chance accuracy across all conditions of the spatial attention task. Of the remaining participants, two older participants completed most (80–90%) of the main task and were included in the analyses. Thus, we analysed the data from 58 younger (25 male, 33 female; median age = 24 years, interquartile range [IQR] = 8) and 61 older (22 male, 39 female; median age = 70 years, IQR = 7) participants. This sample size is sensitive to correlations (e.g., between performance metrics and audiometric thresholds; *N* = 119; power = 0.8) of size *r*^2^ ≥ 0.06, and differences in Pearson’s correlation coefficient (i.e. different relationships between performance and audiometric thresholds in older and younger groups) of size Cohen’s *q* ≥ 0.5.

**Fig. 3:**
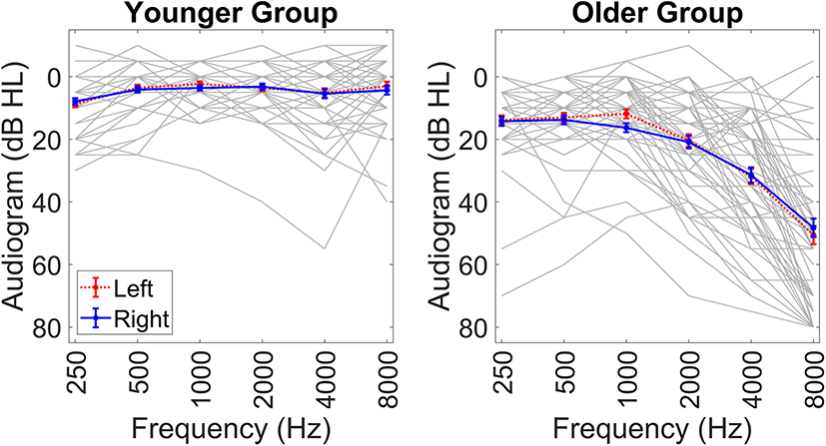
Pure-tone audiometric thresholds for the younger and older groups. Blue and red lines indicate the averages for the right and left ears in each group. Grey lines indicate the averages across both ears for individual participants.

All participants were native English speakers and had normal or corrected-to-normal vision. We did not include participants who reported hearing loss in only one ear. We measured participants’ pure-tone audiometric thresholds at both ears (at octave frequencies between 250 and 8000 Hz) in accordance with BS EN ISO 8253 − 1^80^ using a Starkey Acoustic Analyser AA30 (Starkey Laboratories, Inc.). Audiograms for all participants are illustrated in Fig. 3. The younger group had average pure-tone thresholds of 8.32 dB HL (range = 0–47.5) and the older group had average pure-tone thresholds of 14.06 dB HL (range = -5–30). For all participants, the difference in six-frequency average pure-tone thresholds between the left and right ears was 22.5 dB or less (mean = 6.52 dB, standard deviation = 3.76). Participants who used hearing aids (*N* = 10, all in the older group) removed them while completing the tasks. None of the participants reported previous ear surgery. We did not exclude participants with tinnitus. We did not ask participants about Ménière’s disease.

The study was approved by the UCL Research Ethics Committee (ID: 12929/002). All participants gave informed consent and were compensated for their time at standard UCL rates. All research was performed in accordance with relevant guidelines and regulations.

### Materials and procedures

The experiment was conducted in a sound-attenuating booth. Participants sat in a chair facing an LCD visual display unit (Dell Inc.). All tasks were delivered using custom-written MATLAB scripts (version R2019a; MathWorks, Inc.) with Psychtoolbox^81^ (version: 3.0.17). Acoustic stimuli were presented through an external sound card (ESI Maya 22 USB; ESI Audiotechnik GmbH, Leonberg) connected to circumaural headphones (Sennheiser HD 380 Pro; Sennheiser electronic GmbH & Co. KG). Participants’ responses were recorded using a touch screen (iiyama ProLite T2435MSC; iiyama Corporation).

#### WAIS matrix reasoning task

Participants first completed a computerised version of the Wechsler Adult Intelligence Scale-IV (WAIS-IV) Matrix Reasoning test^52^, which is often used to assess fluid intelligence^49^. On each trial, participants viewed an array of images, which contained one missing image. For example, sometimes they viewed a 2 × 2 array of images in which the lower right image was missing; other times they viewed a 1 × 5 array of images in which the far-right image was missing. On each trial, they were presented with five options of images that could fit into the array, and were instructed to select one image. Before commencing the test, participants were provided with two examples with written explanations.

#### Spatial attention task

Next, participants completed a spatial attention task. Acoustic stimuli were modified phrases from the Co-ordinate Response Measure corpus^82^ (CRM) used by Holmes et al.^22,28^. Each phrase contained a colour word (“Red”, “Green”, “Blue”, or “White”), a number word (“one”, “two”, “three”, or “four”), and the word “now”; for example, “Red two now”. The phrases were spoken by native British English talkers. We wanted to present three distinct voices on each trial; given that age and gender are salient voice cues, we used one male voice, one female voice, and one “child” voice, which included phrases spoken by a different female talker that were manipulated using Praat© (Version 5.3.08; http://www.praat.org/) to have a higher fundamental frequency and shorter formant spacing ratio than the original recordings. The digital recordings had an average duration of 1.4 s and were all adjusted to have the same root mean square (RMS) power. Visual stimuli were white arrows on a black background that pointed leftwards or rightwards (left and right spatial cues, respectively), and a composite stimulus that contained both arrows overlaid.

Figure 1a illustrates the trial structure for the spatial attention task. On every trial, participants saw the visual composite stimulus (which was uninformative about the target location) followed by the left or right spatial cue, which indicated the location of the target talker and varied from trial to trial. There were two cue-target interval conditions: In the longer cue-target interval condition, the composite stimulus was presented for 1000 ms, then the spatial cue was presented for 2000 ms before the acoustic stimuli began; in the shorter cue-target interval condition, the composite stimulus was presented for 2900 ms, then the spatial cue was presented for 100 ms before the acoustic stimuli began. Including the composite stimulus ensured that the length of time between the end of the fixation cross and the start of the acoustic stimuli was equal on every trial; thus, differences between cue-target interval conditions are due to differences in the amount of time that the visual stimulus is informative and not differences in predictability of the onset of acoustic stimuli. In all conditions, the spatial cue remained on the screen until the acoustic stimuli had ended, so participants did not have to maintain the cue in memory. The acoustic stimuli for each trial consisted of three overlaid phrases, which were always spoken by different talkers, contained different colour and number words, and were simulated to come from different spatial locations using ITDs. The child’s voice was always presented with an ITD of 0 µs; in other words, it was simulated to come from a central location and was never the target. The locations of the male and female voices varied pseudo-randomly on each trial. One had an ITD of -205 µs, and was therefore simulated to the left side. The other had an ITD of + 205 µs, and was therefore simulated to the right side. For each trial, the TMR was selected from one of eleven values (-18, -15, -12, -8, -4, 0, + 4, +8, + 12, +15, or + 18 dB), which specified the level difference between the target phrase and each of the two competing phrases. The overall amplitude of the combined phrases was set to the same RMS value across trials. Participants were instructed to listen to the voice that corresponded to the direction of the arrow, and to report the colour-number combination from the target phrase by pressing one of sixteen coloured digits on the touch screen, which were displayed on the touch screen from the beginning of each trial. Participants were instructed to respond as quickly and as accurately as possible, and to guess if uncertain. There was no limit on response time. The intertrial interval was randomly selected on each trial (1.0–1.5 s).

Participants first completed 12 practice trials, which were identical to the main task except that explicit feedback was provided (i.e., “correct” or “incorrect” was displayed on the screen after each trial). They then completed the main task, which consisted of 440 trials without feedback, separated into 10 blocks of 44 trials. Each block contained one trial from each condition (2 cue-target intervals x 2 spatial cue directions x 11 TMRs).

#### ITD discrimination task

After the spatial attention task, participants completed an adaptive ITD discrimination task using a procedure similar to Dai et al.^35^, except that the stimuli were spoken phrases from the spatial attention task rather than complex tones. On each trial, participants heard two phrases spoken one after another. The second phrase was spoken by the “child” and was located centrally (ITD = 0 µs), while the first sentence was spoken by the male or female talker and was either to the left (negative ITD) or right (positive ITD). Participants were asked to report whether the first sentence was to the left or the right by pressing buttons on the touch screen that were labelled “left” and “right”. The buttons were displayed from the beginning of each trial. Participants were instructed to respond as accurately as possible, and told that they did not need to respond quickly. The ITD was manipulated using an 1-up 3-down adaptive procedure, with a starting ITD of 287 µs and an initial step size of 12.8 µs, which decreased to 0.512 µs after 3 reversals. The run ended after the participant reached a maximum of 10 reversals, reached the minimum ITD value of 0.28 µs, or exceeded 192 trials (whichever happened first). The ITD was adapted in separate runs for the male and female voice, and the order of the two runs was counterbalanced across participants.

For each run, we calculated the ITD threshold as the median of the final 6 reversals, then took the average across the two runs for each participant. Three participants reached the minimum ITD value for one run and, for these participants, we used a value of 0 as their ‘threshold’ for that run. Three participants reached the maximum number of trials on both runs, so we excluded them from further analyses involving the ITD threshold (although, we included these participants in other analyses not involving ITD thresholds). For participants who reached the maximum number of trials on either the male (*N* = 13) or the female (*N* = 8) run, we used the other run as their ITD threshold.

### Analyses

Statistical analyses were conducted with MATLAB© (version R2022a, The MathWorks, Inc., Natick, MA, USA) and JASP™ (versions 0.17.2.1^83^ and 0.18.1^84^). We calculated effect sizes and confidence intervals (CIs) using MOTE^85^. All tests are two-tailed, with an alpha of 0.05.

#### Spatial attention task: accuracy

We used a three-way mixed ANOVA to compare the effects of Group (younger or older; between-subjects variable), TMR (11 levels from − 18 to + 18 dB; within-subjects variable), and Cue-Target Interval (2000 or 100 ms; within-subjects variable) on the proportion of correct responses.

We compared the benefit from the longer cue-target interval (ΔAcc) between groups using an independent samples *t*-test.

#### Spatial attention task: reaction times

Reaction times (RTs) were measured from the onset of the acoustic stimuli. Consistent with previous studies, we analysed RTs for correct trials only^86^. We only included RTs at TMRs from − 8 dB to + 18 dB, because many participants had no correct trials below − 8 dB TMR, so including TMRs below − 8 dB TMR could bias the analyses towards the best-performing participants. The cut-off TMR of -8 dB was chosen to ensure that at least 70% of participants in both groups had at least one correct trial in each Cue-Target Interval condition (for comparison, 100% of participants had at least one correct trial per condition at all positive TMRs, but below − 8 dB TMR, ≤ 51% of participants in the older group had at least one correct trial per condition). We used a three-way mixed ANOVA to compare the effects of Group, TMR, and Cue-Target Interval on RTs.

We compared the RT benefit from the longer cue-target interval (ΔRT) between groups using an independent samples *t*-test.

#### Individual differences: relationships among tasks

We used Spearman’s correlation coefficients to examine the relationships among tasks, due to non-normality of residuals and heteroskedasticity in the data, and we used Bonferroni corrections for multiple comparisons. Where we obtained different relationships in the younger and older groups, we performed follow-up bootstrapping analyses (using 10,000 samples with replacement) to compare the Spearman’s correlation coefficients between groups, to test the hypothesis that the two coefficients are drawn from different distributions.

To examine performance, overall, accounting for potential speed-accuracy trade-offs, we also examined relationships with linear integrated speed-accuracy scores (LISAS^87^) on the spatial attention task (see Supplemental File; Supplemental Fig. 1; Supplemental Table 1).

## Electronic supplementary material

Below is the link to the electronic supplementary material.

Supplementary Material 1

## Data Availability

Experiment files, data, and analysis code for this study are publicly available on the Open Science Framework (OSF) and can be accessed at https://osf.io/gvys3/?view_only=f7f27642ef984c3aa10ab046135b8862.
